# Benchmarking of Halogen Bond Strength in Solution with Nickel Fluorides: Bromine versus Iodine and Perfluoroaryl versus Perfluoroalkyl Donors

**DOI:** 10.1002/chem.201900924

**Published:** 2019-06-18

**Authors:** Sarah J. Pike, Christopher A. Hunter, Lee Brammer, Robin N. Perutz

**Affiliations:** ^1^ Department of Chemistry University of York, Heslington York YO10 5DD UK; ^2^ Department of Chemistry University of Cambridge Cambridge CB2 1EW UK; ^3^ Department of Chemistry University of Sheffield, Brook Hill Sheffield S3 7HF UK

**Keywords:** halogen bonds, nickel fluorides, NMR spectroscopy, perfluoroaryl and perfluoroalkyl donors, solution

## Abstract

The energetics of halogen bond formation in solution have been investigated for a series of nickel fluoride halogen bond acceptors; *trans*‐[NiF(2‐C_5_NF_4_)(PEt_3_)_2_] (**A1**), *trans*‐[NiF{2‐C_5_NF_3_(4‐H)}(PEt_3_)_2_] (**A2**), *trans*‐[NiF{2‐C_5_NF_3_(4‐NMe_2_)}(PEt_3_)_2_] (**A3**) and *trans*‐[NiF{2‐C_5_NF_2_H(4‐CF_3_)}(PCy_3_)_2_] (**A4**) with neutral organic halogen bond donors, iodopentafluorobenzene (**D1**), 1‐iodononafluorobutane (**D2**) and bromopentafluorobenzene (**D3**), in order to establish the significance of changes from perfluoroaryl to perfluoroalkyl donors and from iodine to bromine donors. ^19^F NMR titration experiments have been employed to obtain the association constants, enthalpy, and entropy for the halogen bond formed between these donor‐acceptor partners in protiotoluene. For **A2**–**A4**, association constants of the halogen bonds formed with iodoperfluoroalkane (**D2**) are consistently larger than those obtained for analogous complexes with the iodoperfluoroarene (**D1**). For complexes formed with **A2**–**A4**, the strength of the halogen bond is significantly lowered upon modification of the halogen donor atom from I (in **D1**) to Br (in **D3**) (for **D1**: 5≤*K*_285_≤12 m ^−1^, for **D3**: 1.0≤*K*_193_≤1.6 m ^−1^). The presence of the electron donating NMe_2_ substituent on the pyridyl ring of acceptor **A3** led to an increase in −Δ*H*, and the association constants of the halogen bond complexes formed with **D1**–**D3**, compared to those formed by **A1**, **A2** and **A4** with the same donors.

## Introduction

Halogen bonding interactions are rapidly emerging as key constituents of the molecular recognition toolbox.^1^, ^2^, ^3^ The utility and importance of halogen bonding interactions are evident through their widespread use in applications including crystal engineering,^4^ materials chemistry,^5^ supramolecular chemistry,^6^ and anion recognition^7^ and through its emergent role in organocatalysis and reactivity.^8^, ^9^ Halogen bonding interactions are known to hold great significance in medicinal chemistry^10^, ^11^ and are also recognized to be important in achieving function in biological systems.^12^, ^13^, ^14^ The formation of halogen bonding interactions to species in the “ligand domain” has been revealed crystallographically^15^, ^16^ and in solution. (The ligand domain consists of those ligand atoms directly bonded to the metal or with a strong electronic interaction with it.)^16^ There remains, however, a distinct shortage of information about the energetics of these halogen bonding interactions in solution.^17^, ^18^, ^19^ In contrast, the energetics of halogen‐bonded systems involving organic acceptor and donor partners are better documented and include association constants for halogen bonds formed between haloalkynes,^20^ haloarenes^21^ and haloalkanes^22^ as donors with neutral organic bases. Taylor and co‐workers investigated the influence of the type of donor, fluorinated aryl (C_6_F_5_I) and fluorinated alkyl (C_8_F_17_I), on the strength of the halogen bond formed with a wide range of organic bases in cyclohexane at 298 K.^23^ The association constants of the binding event were determined by ^19^F NMR spectroscopy, demonstrating that the equilibrium constants were larger for all the halogen bond donor‐acceptor partners with the iodoperfluoroalkane halogen bond than with the analogous iodoperfluoroarene interaction (e.g., C_6_F_5_I⋅⋅⋅OPBu_3_, 12±2.5 m ^−1^ and C_8_F_17_I⋅⋅⋅OPBu_3_, 18±4 m ^−1^).^23^ Resnati and co‐workers employed ^19^F NMR spectroscopy to identify that changing the halogen donor atom from iodine in 1,2‐diiodotetrafluorobutane to bromine in 1,2‐dibromotetrafluorobutane significantly weakened the halogen bond formed with quinuclidine in hydrocarbon solvents.^24^ The influence of the halogen in perfluoroaryl donors (C_6_F_5_X, where X=Br and I) on the strength of halogen bonds formed with 1,4‐diazabicyclo[2.2.2.]octane (DABCO) has been studied computationally and experimentally.^25^ Halogen‐bond complexes formed between C_6_F_5_I and DABCO were present in [D_8_]toluene, but those formed with C_6_F_5_Br were less prominent and thus weaker, due to competing solvent interactions. Bowling and co‐workers used ^19^F and ^15^N NMR spectroscopy to investigate the formation of intramolecular halogen bonds between a C_6_F_4_X unit (X=I and Br) and a pyridyl moiety (py) in which the halogen bond donor and acceptor units are linked by an aryldiyne spacer. The evidence indicated that the C_6_F_4_Br⋅⋅⋅py interaction is probably significantly weaker than the corresponding C_6_F_4_I⋅⋅⋅py interaction in benzene solution.^26^

We have previously demonstrated that the ^19^F M−F resonance of *trans*‐[MF(py^F^)(PR_3_)_2_] complexes, where M=Ni, Pd or Pt, py^F^=fluorinated 2‐pyridyl and R=ethyl (Et) or cyclohexyl (Cy), is extremely sensitive to chemical environment and can be employed as an NMR spectroscopic probe of the energetics of formation of 1:1 halogen bond adducts between iodopentafluorobenzene and the metal fluoride complexes.^17^ For the most closely related complexes, the enthalpy of dissociation of the halogen bond followed the order: Pt>Pd>Ni.^17^ These studies established that modification of the electronic nature of the substituents on the fluoropyridyl ligand (py^F^), by replacement of one fluorine by hydrogen or CF_3,_ had no significant effect on the thermodynamic data, but the influence of the phosphine ligand was marked. Crystallographic characterization of this class of halogen bonds has been achieved for the closely related self‐complementary nickel fluorides, *trans*‐[NiF(4‐C_6_F_4_I)(PEt_3_)_2_] and *trans*‐[NiF(2‐C_6_F_4_I)(PEt_3_)_2_], in which a chain of molecules is formed, linked by intermolecular I⋅⋅⋅F halogen bonds.^27^ Other authors have shown that 1:1 halogen bonded complexes with C_6_F_5_I are also formed by nickel fluoride complexes with pincer ligands, and by fluoride complexes of zinc and magnesium.^18^ The formation of halogen‐bonding interactions in the ligand domain of metal complexes is not restricted to metal halides; a series of bis(*η*‐cyclopentadienyl)metal hydrides have been shown to form halogen bonds with C_6_F_5_I in toluene,^19^ and metal cyanides have been identified as halogen bond acceptors crystallographically.^28^

**Scheme 1:**
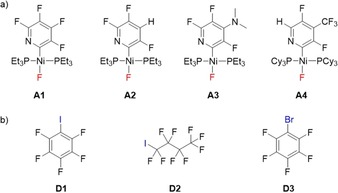
a) Metal fluorides **A1**–**A4** employed as halogen bond acceptors in this study. b) halogen bond donors **D1**–**D3**.

Reports on the energetics of halogen bonding in solution mainly focus on the use of iodinated donors, whereas brominated donors feature less frequently,28a owing to the weaker halogen bonds formed with this donor atom,^29^ which renders acquisition of solution‐based data more challenging.^30^ Until now, we also lacked information about the behavior of iodoperfluoroalkane donors towards transition metal fluorides. In this paper, we describe a systematic study of the influence of structural variations of the donor and acceptor species on the binding constants and energetics of halogen bond formation between a series of structurally related nickel fluorides **A2**–**A4** and a range of organic iodo‐ and bromo‐perfluorocarbon donors **D1**–**D3** in protiotoluene (Scheme 1). The halogen bond donors are iodopentafluorobenzene (the standard), iodononafluorobutane and bromopentafluorobenzene. The halogen bond acceptors maintain the square planar nickel fluoride geometry, but vary the substituents on the pyridyl ring and, in **A4** the phosphine ligand. Although **A4** represents a change in the two parameters, both the pyridyl ring and phosphine ligand, we have previously shown that substitution of F by CF_3_ on the pyridyl ring had little effect on the energetics of halogen bond formation. The results provide a benchmark for these halogen bond donors, which are in common use in supramolecular assemblies directed by halogen bonding.

## Results and Discussion

**Figure 1:**
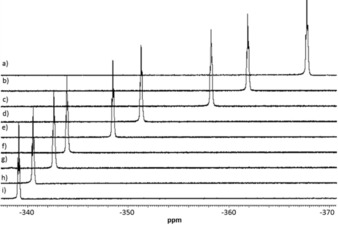
Stack plot of ^19^F NMR spectra in the nickel fluoride region (toluene‐h_8_, 285 K), at different molar ratios of [**D2**]/[**A3**]. Molar ratios a) 0, b) 0.6, c) 1.0, d) 2.3, e) 3.3, f) 5.9, g) 7.1, h) 10, i) 15.3.

Nickel fluorides were employed as halogen bond acceptors as they are soluble in toluene and do not display appreciable self‐association.17a We reported the formation of the halogen‐bonded adduct **D1⋅A1** and **D1⋅A4** earlier.^17^ **A2**–**A4** were prepared according to known literature procedures.^17^, ^31^ The study of **A2**–**A4** permits investigation of the influence of the substitution pattern of the fluoropyridyl ring on the energetics of the halogen bond formed with a range of organic donors (**D1**–**D3**) (Scheme 1). Accordingly, a series of ^19^F NMR titration experiments were performed on metal fluorides (**A2**–**A4**) through the addition of increasing quantities of halogen bond donors (**D1**–**D3**) in protiotoluene. The ^19^F resonances of the fluoride ligand directly bound to the metal center in the adducts of **A1**–**A4** appear at high field (e.g., *δ*=−339.3 ppm for **A3** at 285 K, Figure 1(i)) and no overlap with other signals occurs during the titration experiments.

**Figure 2:**
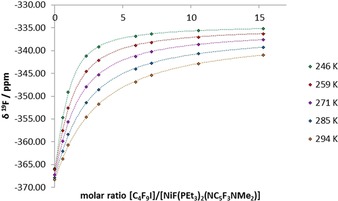
Titration curves at 246, 259, 271, 285, and 294 K for **D2** and **A3** in toluene‐h_8_, showing *δ*(^19^F) of the metal fluoride vs. [**D2**]/[**A3**]. [**A3**]=17 mmol dm^−3^. Diamonds are experimental points; dashed line shows best fit to a 1:1 binding isotherm.

The NMR titration experiments show only one ^19^F resonance as the halogen‐bond adduct is in fast exchange on the NMR timescale. Upon the addition of **D1** or **D2**, the ^19^F NMR signal of the metal fluoride in **A2**–**A4** exhibited a marked downfield shift with *δ* rising by ca. 20–30 ppm at 285 K upon treatment with a large excess of either perfluoroiodine donor (for example, **A2** *δ*=−367.9 and for **D2⋅A3** *δ*=−339.3, see Figure 1). This shifting behavior is attributed to formation of the halogen bond adduct.^17^ As expected for an equilibrium between monomers and an intermolecular complex, the chemical shifts increase at lower temperature as the equilibrium is shifted towards the adduct (Figure 2). For titrations of **D3** against **A2**–**A4**, the changes in the spectra are negligible in the temperature range used for the **D1** and **D2**. For this reason, experiments were conducted at 193 K in order to shift the equilibria towards the halogen bonded adduct, but the change in chemical shift was significantly smaller than for **D1** and **D2** at 8–10 ppm (see Supporting Information). Through fitting the variation of the chemical shift of the ^19^F NMR resonance with the molar ratio of [donor]/[acceptor], association constants for the halogen bonding interaction were obtained by titrations for **D2**–**3⋅A2**–**4**. For all systems, the titration data fit well to a 1:1 binding isotherm (Figure 2 and Supporting Information) as in Equation (1):(1)
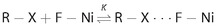



in which R=C_6_F_5_ or C_4_F_9_ and X=I or Br.

**Figure 3:**
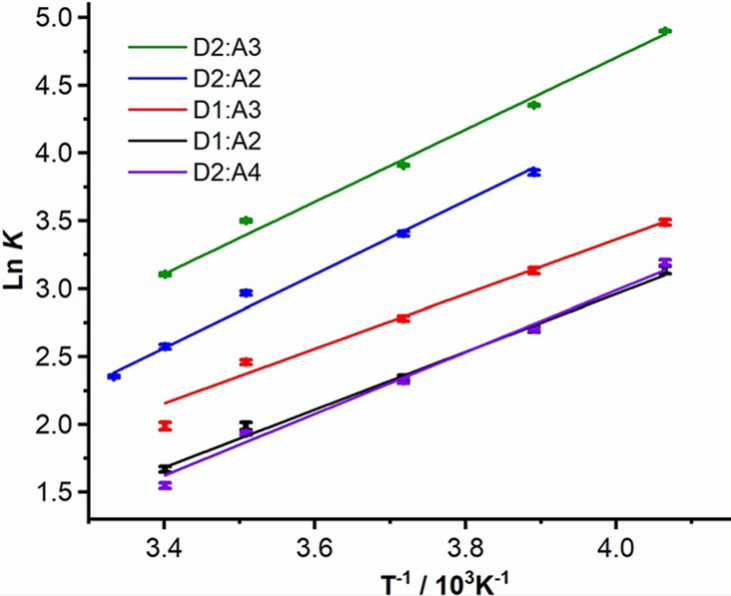
Van't Hoff plots for halogen‐bonded pairs **D1⋅A2**, **D2⋅A2**, **D1⋅A3**, **D2⋅A3** and **D2⋅A4**.

There are two parameters to be fitted: the equilibrium constant *K* and the downfield shift from the signal of the free fluoride for the coordinated fluoride of the adduct, Δ*δ*_19F_.^32^ The fitting routine for each titration curve models the chemical shift difference, Δ*δ*_fit_, between the free metal complex and the halogen‐bonded adduct. The value of Δ*δ*_fit_ lies between 31 and 38 ppm at 285 K for **A2**–**A4** with iodinated donors **D1** or **D2** and between 13 and 19 ppm for **A2**–**A4** with brominated donor **D3** at 193 K (Table 1). The change in the chemical shift (Δ*δ*) of the ^19^F resonance observed experimentally correlates well with the calculated Δ*δ*_fit_ values (Supporting Information). The values of Δ*δ*_fit_ varied with temperature by no more than 1.1 ppm. From the experimental titration data, both the standard enthalpy and entropy of the halogen bonding interactions between **A2**–**A4** and **D1** and between **A2**–**A4** and **D2** were calculated from Van't Hoff plots. Analysis of the titration data gave excellent fits with correlation coefficients *R* ^2^>0.975 for all systems studied (Figure 3 and Supporting Information). The thermodynamic parameters and association constants for all experiments are reported in Table 1.

**Table 1:** Summary of thermodynamic parameters for halogen bonding of donors **D1**–**D3** with nickel fluorides **A1**–**A4** in protiotoluene.^[a]^

Donor	Acceptor	Δ*H*° [kJ mol^−1^]	Δ*S*° [J mol^−1^ K^−1^]	*K*_285_ [m ^−1^]	Δ*δ* ^fit^_285 K_	*R* ^2^
**D1**	**A1**	−16±1^[b]^	−42±4^[b]^	5.5±0.1^[b]^	33.4^[c]^	–
**D1**	**A2**	−18±2	−46±8	7.1±0.2	32.6	0.995
**D1**	**A3**	−17±5	−39±19	11.3±0.2	33.5	0.975
**D1**	**A4**	−19±4^[d]^	−54±1^[d]^	4.4±0.2^[d]^	35.6^[e]^	0.998^[c]^
**D2**	**A2**	−23±4	−53±14	18.6±0.3	31.4	0.993
**D2**	**A3**	−22±3	−50±12	31.8±0.3	32.0	0.994
**D2**	**A4**	−19±4	−51±13	6.9±0.1	38.0	0.990
**D3**	**A2**	–	–	1.0±0.1^[f]^	17.7^[f]^	–
**D3**	**A3**	–	–	1.6±0.1^[f]^	12.5^[f]^	–
**D3**	**A4**	–	–	1.3±0.1^[f]^	18.9^[f]^	–

[a] Errors at 95 % confidence level. Δ*δ*=Chemical shift difference between free metal fluoride, Ni−F, and R−X⋅⋅⋅F−Ni adduct calculated by the fitting routine. [b] From Ref. 17a. [c] At 289 K from Ref. 17a. [d] From Ref. 17b. [e] At 303 K from Ref. 17b. [f] Determined at 193 K.

**Figure 4:**
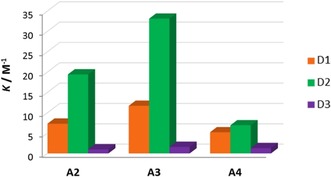
Variation in *K* with donor and acceptor. Measurements at 285 K for **D1** and **D2** but 193 K for **D3**.

The presence of the strongly electron‐donating group, NMe_2_, at the 4‐position of the pyridyl ring in **A3** leads to larger *K* values for **D1⋅A3** and **D2⋅A3** than for analogous adducts formed with **A2**, which bear a hydrogen at the same position on the ring (Table 1 and Figure 4). The PCy_3_ complex, **A4**, forms halogen bonds with the iodoperfluorocarbon donors, **D1** and **D2**, that have lower *K* values than with any of the PEt_3_ bearing complexes **A1**–**A3** (Table 1). The electronic nature of the donor influences the strength of the interaction with complexes following the order **D2**>**D1**≫**D3** (Table 1).^23^, ^33^ Use of the iodoperfluoroalkane donor **D2** results in considerably larger equilibrium constants than those for iodopentafluorobenzene,1d, 19, 23, 30 whereas modification of the halogen donor atom from iodo‐ to bromo‐ in the perfluoroarenes **D1** and **D3** greatly reduces the strength of the interaction of the halogen bond adduct formed (Table 1).^24^, ^26^, ^34^ The titration data also show a significant reduction in the magnitude of chemical shift change of the ^19^F resonance for **D3⋅A*n*** versus **D1⋅A*n*** (*n*=1–4). The association constants obtained for the halogen bonding interaction with **D3** were all recorded at a single temperature (193 K), as above this temperature the binding constant was too low to be measured accurately. The differences between *K*_193_ values for **D3** with different acceptors are small.

The halogen bonding interactions of **D1** with **A1**–**A4** and **D2** with **A2**–**A4** have favorable enthalpic terms and unfavorable entropic terms, (**D1**: −19≤Δ*H*°≤−16 kJ mol^−1^ and −54≤Δ*S*°≤−39 J K^−1^ mol^−1^ and **D2**: −23 ≤ Δ*H*° ≤ −19 kJ mol^−1^ and −53≤Δ*S*°≤−50 J K^−1^ mol^−1^) in line with literature reports.^17^ The enthalpic contribution for the **D2⋅A2** complex is larger than for **D1⋅A2**, with a difference that is just significant at the 95 % confidence level. The Δ*H*° and Δ*S*° terms of the halogen bonding interactions of **A2** and **A3** are each comparable for both the aromatic donor **D1**,17b and aliphatic donor **D2**, showing that the changes in the energetics on introducing the NMe_2_ substituent on the fluoropyridyl ring are too small to identify the source of the effect. The Δ*H*° and Δ*S*° terms for the halogen bonding interactions formed between donors **D1** and **D2** and acceptor **A4** are within error of the analogous interactions formed with **A1**–**A3**, despite the smaller binding constants for the former. As only one temperature was employed to study the halogen bond formation of **D3** with **A2**–**A4**, the enthalpic and entropic terms could not be calculated for this interaction.

## Conclusion

The abilities of a series of structurally related nickel fluorides, *trans*‐[NiF{2‐C_5_NF_2_H(4‐CF_3_)}(PEt_3_)_2_] (**A2**), *trans*‐[NiF{2‐NC_5_F_3_(4‐NMe_2_)}(PEt_3_)_2_] (**A3**) and *trans*‐[NiF{2‐C_5_NF_2_H(4‐CF_3_)}(PCy_3_)_2_] (**A4**), to accept halogen bonds from a range of organic halogen‐bond donors, iodopentafluorobenzene (**D1**), 1‐iodononafluorobutane (**D2**) and bromopentafluorobenzene (**D3**) in protiotoluene have been established using a series of ^19^F NMR titration experiments. These measurements supplement previous studies of *trans*‐[NiF{2‐C_5_NF_4_}(PEt_3_)_2_] (**A1**) and *trans*‐[NiF(2‐C_5_NF_4_)(PCy_3_)_2_] with **D1**. Binding constants have been determined for the interactions between **D1**–**D3** and **A2**–**A4**. Enthalpies and entropies of halogen bond formation between iodinated halogen bond donors and nickel fluoride acceptors have been determined.

For halogen bonds formed with **A1**–**A4**, the aliphatic donor **D2** has association constants close to three times greater than those observed for the aromatic donor **D1**, which is in accordance with the relative strengths of the two donors observed in halogen bonds with organic acceptors and transition metal hydrides.^19^ There is a corresponding and consistent increase in −Δ*H*°. The association constants for the halogen bond interaction with **D1** are significantly higher than those observed with the brominated analogue **D3**, which reflects the markedly different donor capabilities of the halogen atoms, I and Br. These observations are in line with reports of corresponding complexes formed with DABCO,^25^ and correlate with studies of intramolecular halogen bonding.^26^ The introduction of an NMe_2_ electron‐donor group on the fluoropyridyl ring results in a marked increase in association constant.

This investigation provides the first determination of energetics of halogen bond formation for aliphatic donors and for bromine donors with metal‐fluoride acceptors. These findings emphasize the utility of metal‐fluorides in providing a benchmark for strengths of halogen bonds to metal complexes and allow comparisons to the strengths of corresponding hydrogen bonds. As for organic systems, the strongest halogen bonds are formed with an iodoperfluoroalkane donor. We anticipate that this study could also play an important role in the future design of synthetic supramolecular systems which exploit halogen bonding interactions.

## Conflict of interest

The authors declare no conflict of interest.

## Supporting information

As a service to our authors and readers, this journal provides supporting information supplied by the authors. Such materials are peer reviewed and may be re‐organized for online delivery, but are not copy‐edited or typeset. Technical support issues arising from supporting information (other than missing files) should be addressed to the authors.

Supplementary
