# Supplementary material for: Benchmarking of Halogen Bond Strength in Solution with Nickel Fluorides: Bromine versus Iodine and Perfluoroaryl versus Perfluoroalkyl Donors
Source: Chemistry. 2019 Jun 18;25(39):9237–41. doi: 10.1002/chem.201900924 (PMC6771525; doi:10.1002/chem.201900924)
Supplement: Supplementary file 1 — Supplementary [file CHEM-25-9237-s001.pdf]

CHEMISTRY
A European Journal

Supporting Information

Benchmarking of Halogen Bond Strength in Solution with Nickel Fluorides: Bromine versus Iodine and Perfluoroaryl versus Perfluoroalkyl Donors

Sarah J. Pike,[a] Christopher A. Hunter,*[b] Lee Brammer,*[c] and Robin N. Perutz*[a]

chem_201900924_sm_miscellaneous_information.pdf

Supporting Information

[†]Department of Chemistry, University of York, Heslington, York, YO10 5DD, UK.

[‡]Department of Chemistry, University of Cambridge, Cambridge, CB2 1EW, UK.

[§]Department of Chemistry, University of Sheffield, Brook Hill, Sheffield, S3 7HF, UK.

Email: herchelsmith.orgchem@ch.cam.ac.uk, lee.brammer@sheffield.ac.uk,

robin.perutz@york.ac.uk

Contents *S2*

General Experimental Section *S3*

Standard Method for Titrations *S3*

Fitting of Titration Curves for Nickel-Fluorides *S4*

1. Table for Composition of Stock Solutions *S5*

2. Titration Curves for $\Delta\delta$ (^{19}F) / ppm *vs* molar ratio [RX] / [Ni-F] *S6*

3. Equilibrium Constants and $\Delta\delta_{fit}$ obtained from macro for titrations *S14*

4. Stack Plots of Titration Data *S17*

5. Summary of Thermodynamic Parameters for Halogen Bonding Interactions *S24*

6. Table of NMR Temperature Calibration *S25*

7. References *S25*

General Experimental Section

All preparations were performed under argon using a glove box or Schlenk line techniques. Iodopentafluorobenzene (**D1**), 1-iodononafluorobutane (**D2**) and bromopentafluorobenzene (**D3**) were degassed and dried over 3 Å molecular sieves. Solvents were dried and distilled over sodium and degassed prior to use. All chemicals and reagents were purchased from Sigma-Aldrich unless otherwise stated. Iodopentafluorobenzene and bromopentafluorobenzene were purchased from Fluorochem Ltd. NMR spectra were collected on a Bruker AMX 500 MHz spectrometer. The temperature of the NMR spectrometer was calibrated by using established methods.[1] Complexes **A1**-**A4** were synthesised according to literature procedures[2,3] or taken from laboratory stock; *trans*-[NiF(2-NC_5F_4)(PEt_3)$_2$] (**A1**)**,** *trans*-[NiF(2-NC_5F_3H)(PEt_3)$_2$] (**A2**), *trans*-[NiF{2-NC_5F_3(4-NMe_2)}(PEt_3)$_2$] (**A3**) and *trans*-[NiF(2-$NC_5F_2HCF_3$)(PCy_3)$_2$] (**A4**), The following abbreviations are employed: Cy = cyclohexyl, Et = ethyl and Me = methyl.

Standard Method for Titrations

Stock solutions were prepared by recording the masses of the host, guest and solvent added. To Young's NMR tap tubes in a glove box were added approximately 450 μL of host stock solution and the exact mass recorded to allow for determination of the number of moles of host in the sample. Similarly, the guest was added by syringe and the mass of the addition recorded. To standardise the volume of the samples, solvent was added to give a volume of 550 μL and the mass of the addition recorded. For the measurement, the samples were kept in a cold bath at approximately the temperature of the spectrometer and allowed two minutes to equilibrate after insertion.

Fitting of titration curves for nickel fluorides

Fitting of titration curves for nickel fluorides: The system involves the formation of a 1:1 adduct between the guest and the nickel fluoride species, which takes place by R–X···F–Ni halogen bonding (where X = Br or I). In the scheme R–X is the halogen bond donor **D1-D3** and F–Ni is the halogen bond acceptor **A1-A4**.

$$\text{R–X} \quad + \quad \text{F–Ni} \quad \underset{}{\overset{K_{eq}}{\rightleftharpoons}} \quad \text{R–X····F–Ni (R} = C_6F_5 \text{ or } C_4F_9\text{, X = I or Br)} \quad \text{(eq 1)}$$

There are two parameters to be fitted: the equilibrium constant K and the downfield shift from the signal of the free fluoride for the coordinated fluoride of the halogen-bonded adduct, $\Delta\delta_{19F}$. The two parameters can be fitted for the whole range of temperatures without any restraints by using a Microsoft Excel macro programmed by Professor Christopher Hunter. ΔH^0 and ΔS^0 were determined by linear regression from the Van't Hoff plots of the equilibrium constants.

Table S1. Composition of stock solutions used for the preparation of samples for halogen bonding measurements by ^{19}F NMR spectroscopy.

	Metal fluoride (host) stock solution		Halogen bond donor (guest) stock solution	
Nickel fluoride / halogen bond donor	mass (Ni-F) / mg	mass (solvent) / mg	mass (Guest) / mg	mass (solvent) / mg
trans-[NiF{2-NC$_5$F$_3$(4-NMe$_2$)}(PEt$_3$)$_2$] (***A3***) / *C$_4$F$_9$I* (***D2***)	50.1	4289.3	290.1	173.3
trans-[NiF(2-NC$_5$F$_3$H)(PEt$_3$)$_2$] (***A2***) / *C$_4$F$_9$I* (***D2***)	49.5	4288.3	307.0	179.1
trans-[NiF(2-NC$_5$F$_3$H)(PEt$_3$)$_2$] (***A2***) / *C$_6$F$_5$Br* (***D3***)	18.5	4312.3	/	/
trans-[NiF(2-NC$_5$F$_2$HCF$_3$)(PCy$_3$)$_2$] (***A4***) / *C$_6$F$_5$Br* (***D3***)	22.2	3573.4	/	/
trans-[NiF(2-NC$_5$F$_3$H)(PEt$_3$)$_2$] (***A2***) / *C$_6$F$_5$I* (***D1***)	41.9	4365	293.8	97.6
trans-[NiF{2-NC$_5$F$_3$(4-NMe$_2$)}(PEt$_3$)$_2$] (***A3***) / *C$_6$F$_5$I* (***D1***)	49.9	3980.7	296.0	98.6
trans-[NiF(2-NC$_5$F$_2$HCF$_3$)(PCy$_3$)$_2$] (***A4***) / *C$_4$F$_9$I* (***D2***)	51.7	4730.8	174.6	209.5
trans-[NiF{2-NC$_5$F$_3$(4-NMe$_2$)}(PEt$_3$)$_2$ (***A3***) / *C$_6$F$_5$Br* (***D3***)	21.6	4374.0	/	/

2. Titration Curves for Δδ (^{19}F) / ppm *vs* molar ratio [RX] / [Ni-F]

*2.1. Titration curves for trans-[NiF(2-NC$_5$F$_3$H)(PEt$_3$)$_2$] (**A2**) with C$_6$F$_5$I (**D1**)*

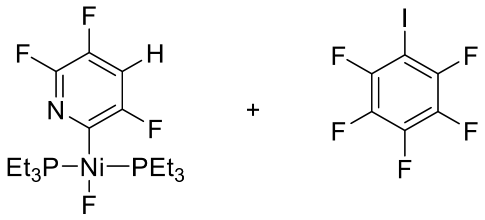

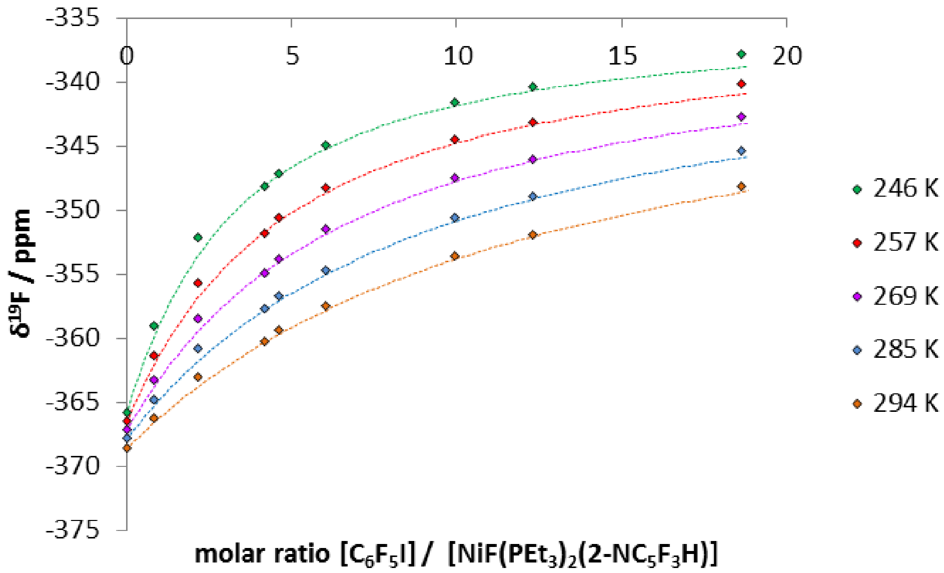

Figure S1 . Fit of the titration curves at different temperatures, showing observed values for δ^{19}F *vs*. ratio of molar concentrations of C$_6$F$_5$I and *trans*-[NiF(2-NC$_5$F$_3$H)(PEt$_3$)$_2$]. (Concentration of *trans*-[NiF(2-NC$_5$F$_3$H)(PEt$_3$)$_2$] = 16 mmol dm^{-3}).

2.2. Titration curves for trans-[NiF{2-NC_5F_3(4-NMe_2)}$(PEt_3)_2$] (**A3**) *with C_6F_5I (**D1**)*

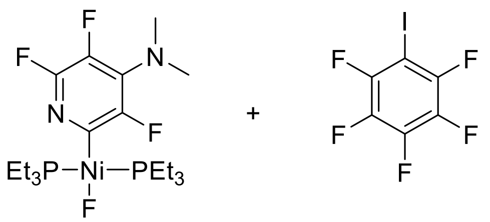

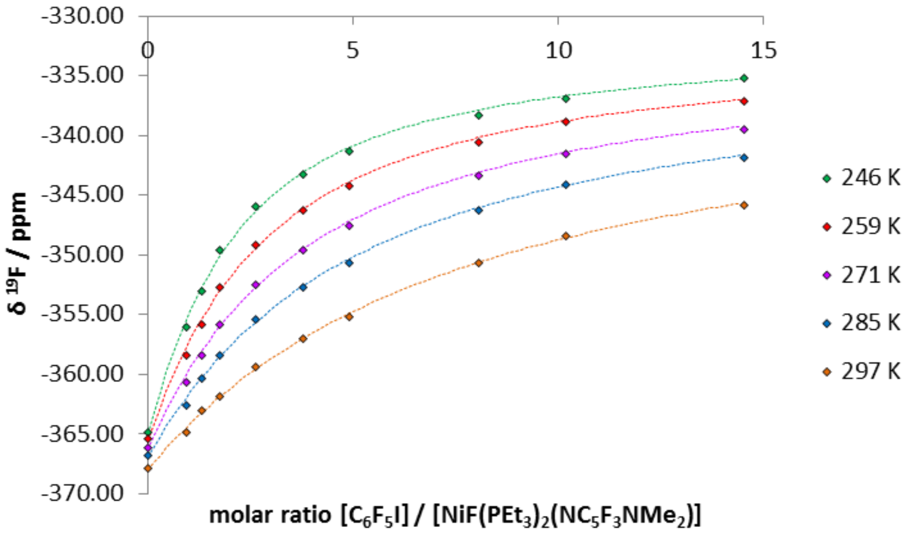

Figure S2. Fit of the titration curves at different temperatures, showing observed values for $\delta^{19}F$ *vs.* ratio of molar concentrations of C_6F_5I and *trans*-[NiF{2-NC_5F_3(4-NMe_2)}$(PEt_3)_2$]. (Concentration of *trans*-[NiF{2-NC_5F_3(4-NMe_2)}$(PEt_3)_2$] = 19 mmol dm^{-3}).

*2.3. Titration curves for trans-[NiF(2-NC$_5$F$_3$H)(PEt$_3$)$_2$] (**A2**) with C$_4$F$_9$I (**D2**)*

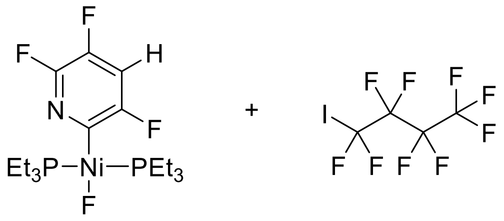

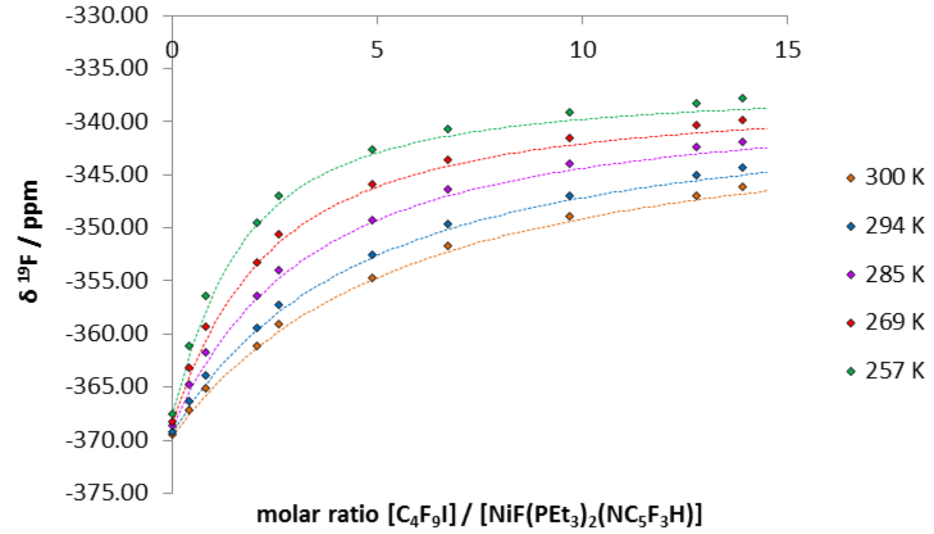

Figure S3. Fit of the titration curves at different temperatures, showing observed values for δ^{19}F *vs*. ratio of molar concentrations of C$_4$F$_9$I and *trans*-[NiF(2-NC$_5$F$_3$H)(PEt$_3$)$_2$]. (Concentration of *trans*-[NiF(2-NC$_5$F$_3$H)(PEt$_3$)$_2$] = 19 mmol dm^{-3}).

*2.4. Titration curves for trans-[NiF{2-NC$_5$F$_3$(4-NMe$_2$)}(PEt$_3$)$_2$] (**A3**) with C$_4$F$_9$I (**D2**)*

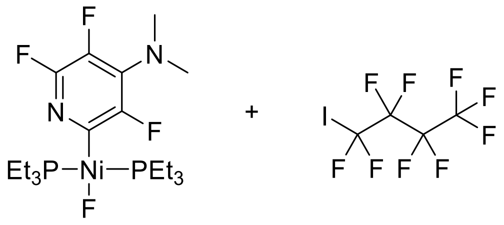

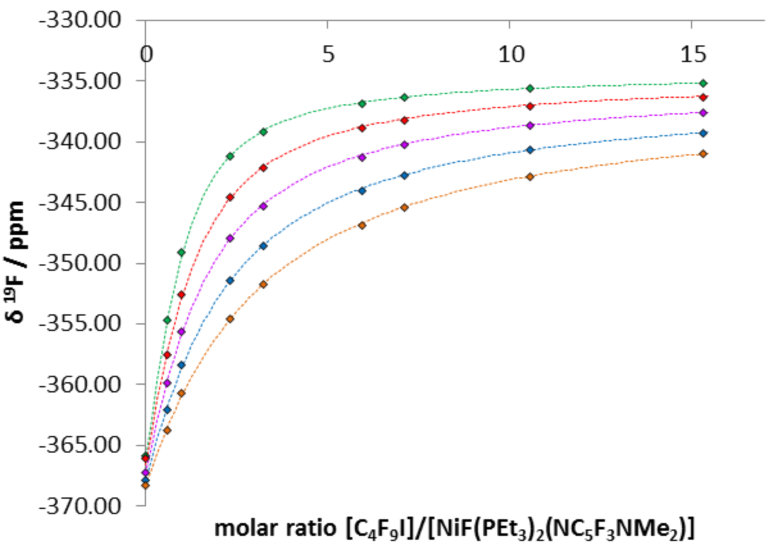

Figure S4. Fit of the titration curves at different temperatures, showing observed values for δ^{19}F *vs.* ratio of molar concentrations of C$_4$F$_9$I and *trans*-[NiF{2-NC$_5$F$_3$(4-NMe$_2$)}(PEt$_3$)$_2$]. (Concentration of *trans*-[NiF{2-NC$_5$F$_3$(4-NMe$_2$)}(PEt$_3$)$_2$] = 17 mmol dm^{-3}).

*2.5. Titration curves for trans-[NiF(2-$NC_5F_2HCF_3$)(PCy_3)$_2$] (**A4**) with C_4F_9I (**D2**)*

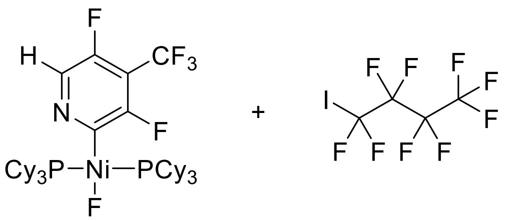

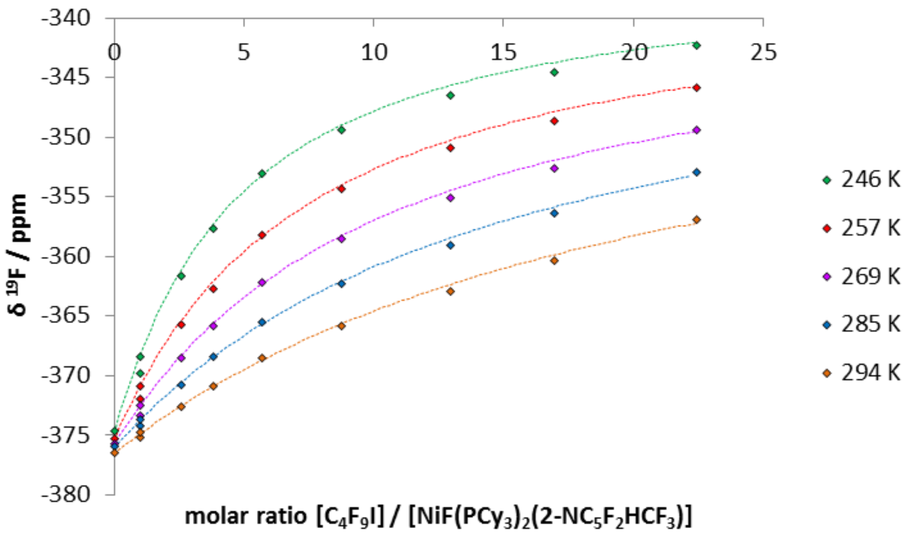

Figure S5. Fit of the titration curves at different temperatures, showing observed values for $\delta^{19}F$ *vs.* ratio of molar concentrations of C_4F_9I and *trans*-[NiF(2-$NC_5F_2HCF_3$)(PCy_3)$_2$]. (concentration of *trans*-[NiF(2-$NC_5F_2HCF_3$)(PCy_3)$_2$] = 10 mmol dm^{-3}).

*2.6. Titration curve for trans-[NiF(2-NC$_5$F$_3$H)(PEt$_3$)$_2$] (**A2**) with C$_6$F$_5$Br (**D3**)*

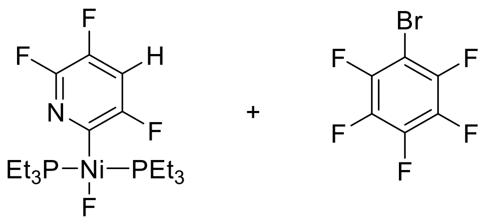

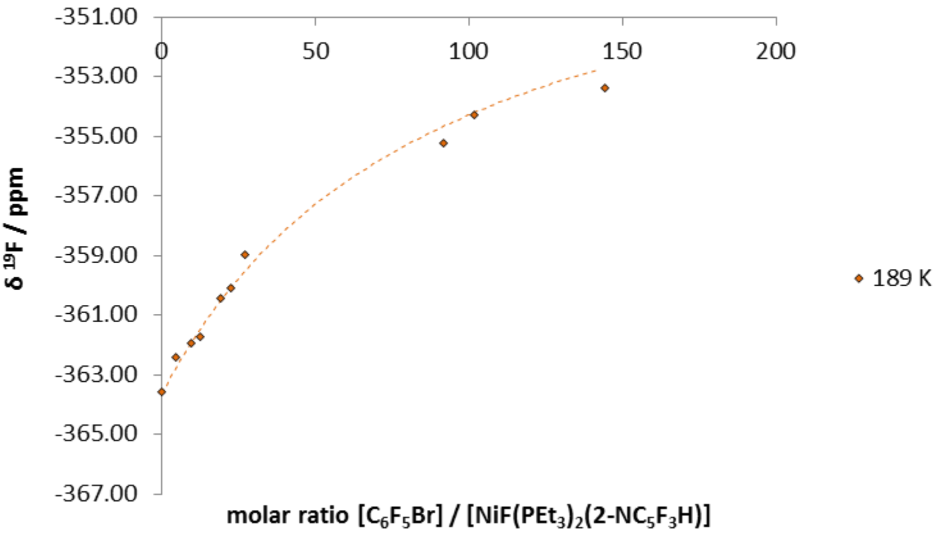

Figure S6. Fit of the titration curve at 193 K, showing observed values for δ^{19}F *vs.* ratio of molar concentrations of C_5F_5Br and *trans*-[NiF(2-NC$_5$F$_3$H)(PEt$_3$)$_2$]. (Concentration of *trans*-[NiF(2-NC$_5$F$_3$H)(PEt$_3$)$_2$] = 7 mmol dm^{-3}).

*2.7. Titration curve for trans-[NiF(2-NC_5F_3(4-NMe_2))(PEt_3)$_2$] (**A3**) with C_6F_5Br (**D3**)*

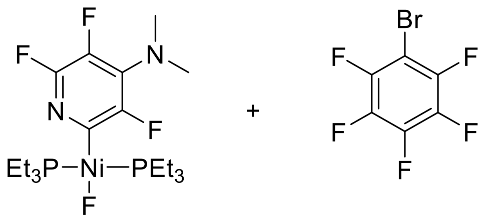

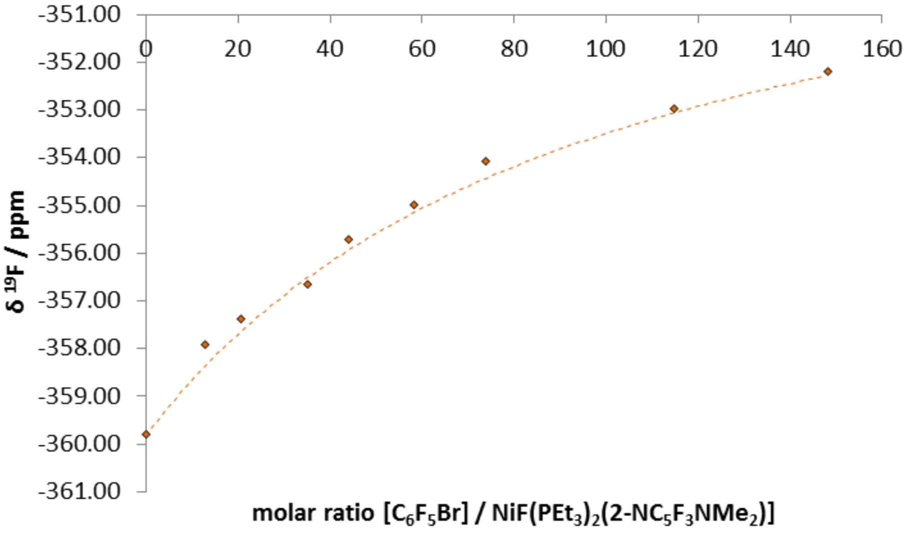

Figure S7. Fit of the titration curve at 193 K, showing observed values for δ^{19}F *vs.* ratio of molar concentrations of C_5F_5Br and *trans*-[NiF{2-NC_5F_3(4-NMe_2)}(PEt_3)$_2$]. (Concentration of *trans*-[NiF{2-NC_5F_3(4-NMe_2)}(PEt_3)$_2$] = 7 mmol dm^{-3}).

*2.8. Titration curve for trans-[NiF(2-NC$_5$F$_2$HCF$_3$)(PCy$_3$)$_2$] (**A4**) with C$_6$F$_5$Br (**D3**)*

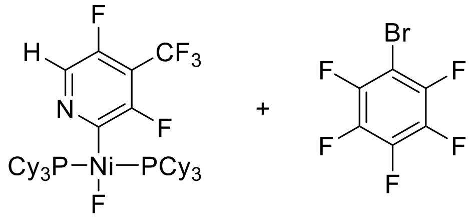

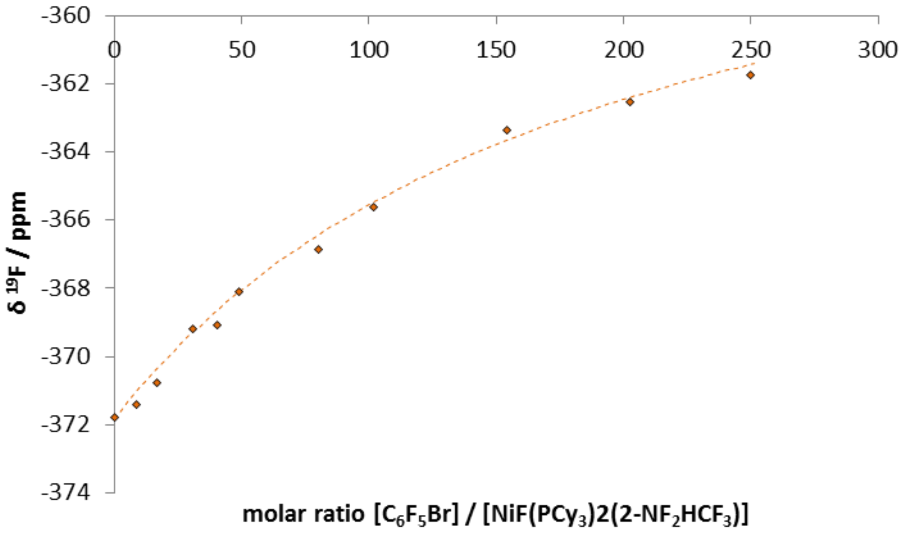

Figure S8. Fit of the titration curve at 193 K, showing observed values for δ^{19}F *vs.* ratio of molar concentrations of C$_5$F$_5$Br and *trans*-[NiF(2-NC$_5$F$_2$HCF$_3$)(PCy$_3$)$_2$].(Concentration of *trans*-[NiF(2-NC$_5$F$_2$HCF$_3$)(PCy$_3$)$_2$] = 3.8 mmol dm^{-3}).

3. Equilibrium Constants and $\Delta\delta_{fit}$ obtained from fit to 1:1 binding isotherm

*3.1. Titration of C_6F_5I (**D1**) against trans-[NiF(2-NC_5F_3H)(PEt_3)$_2$] (**A2**)*

Table S2. Equilibrium Constants and $\Delta\delta_{fit}$ from fit to1:1 binding isotherm for titration of C_6F_5I against *trans*-[NiF(2-NC_5F_3H)(PEt_3)$_2$].

	**246 K**	**257 K**	**269 K**	**285 K**	**294 K**
K (M^{-1})	23.0 ± 0.6	14.9 ± 0.3	10.4 ± 0.2	7.3 ± 0.2	5.3 ± 0.1
**$\Delta\delta_{fit}$** (ppm)	31.1	31.6	32.0	32.6	33.2

*3.2. Titration of C_6F_5I (**D1**) against trans-[NiF{2-NC_5F_3(4-NMe_2)}(PEt_3)$_2$] (**A3**)*

Table S3. Equilibrium Constants and $\Delta\delta_{fit}$ from fit to1:1 binding isotherm for titration of C_6F_5I against *trans*-[NiF{2-NC_5F_3(4-NMe_2)}(PEt_3)$_2$].

	**246 K**	**257 K**	**269 K**	**285 K**	**298 K**
K	32.7 ± 0.7	22.9 ± 0.5	16.1 ± 0.3	11.7 ± 0.2	7.3 ± 0.2
**$\Delta\delta_{fit}$**	33.1	33.3	33.5	33.5	34.0

*3.3. Titration of C_4F_9I (**D2**) against trans-[NiF(2-NC_5F_3H)(PEt_3)$_2$] (**A2**)*

Table S4. Equilibrium Constants and $\Delta\delta_{fit}$ from fit to1:1 binding isotherm for titration of C_4F_9I against *trans*-[NiF(2-NC_5F_3H)(PEt_3)$_2$].

	**257 K**	**269 K**	**285 K**	**294 K**	**300 K**
K	47.2 ± 0.9	30.1 ± 0.5	19.4 ± 0.3	13.1 ± 0.2	10.5 ± 0.1
**$\Delta\delta_{fit}$**	31.2	31.2	31.4	31.6	31.5

*3.4. Titration of C_4F_9I (**D2**) against trans-[NiF{2-NC_5F_3(4-NMe_2)}(PEt_3)$_2$] (**A3**)*

Table S5. Equilibrium Constants and $\Delta\delta_{fit}$ from fit to1:1 binding isotherm for titration of C_4F_9I against *trans*-[NiF{2-NC_5F_3(4-NMe_2)}(PEt_3)$_2$].

	**246 K**	**257 K**	**269 K**	**285 K**	**294 K**
K	134 ± 0.5	77.6 ± 0.5	49.8 ± 0.3	33.1 ± 0.3	22.3 ± 0.2
**$\Delta\delta_{fit}$**	31.9	31.9	32.0	32.0	32.1

*3.5. Titration of C_4F_9I (**D2**) against trans-[NiF(2-$NC_5F_2HCF_3$)(PCy_3)$_2$] (**A4**)*

Table S6. Equilibrium Constants and $\Delta\delta_{fit}$ from fit to1:1 binding isotherm for titration of C_4F_9I against *trans*-[NiF(2-$NC_5F_2HCF_3$)(PCy_3)$_2$].

	**246 K**	**257K**	**269 K**	**285 K**	**294 K**
K	24.3 ± 0.5	14.9 ± 0.2	10.2 ± 0.2	6.9 ± 0.1	4.7 ± 0.1
**$\Delta\delta_{fit}$**	38.5	38.6	38.0	38.0	38.2

*3.6. Titration of C_6F_5Br (**D3**) against trans-[NiF(2-NC_5F_3H)(PEt_3)$_2$] (**A2**)*

Table S7. Equilibrium Constants and $\Delta\delta_{fit}$ from fit to1:1 binding isotherm for titration of C_6F_5Br against *trans*-[NiF(2-NC_5F_3H)(PEt_3)$_2$].

	**193 K**
K	1.0 ± 0.1
**$\Delta\delta_{fit}$**	17.7

*3.7. Titration of C_6F_5Br (**D3**) against trans-[NiF{2-NC_5F_3(4-NMe_2)}(PEt_3)$_2$] (**A3**)*

Table S8. Equilibrium Constants and $\Delta\delta_{fit}$ from fit to1:1 binding isotherm for titration of C_6F_5Br against *trans*-[NiF{2-NC_5F_3(4-NMe_2)}(PEt_3)$_2$].

	**193 K**
K	1.6 ± 0.1
**$\Delta\delta_{fit}$**	12.5

*3.8. Titration of C_6F_5Br (**D3**) against trans-[NiF(2-$NC_5F_2HCF_3$)(PCy_3)$_2$] (**A4**)*

Table S9. Equilibrium Constants and $\Delta\delta_{fit}$ from fit to1:1 binding isotherm for titration of C_6F_5Br against *trans*-[NiF(2-$NC_5F_2HCF_3$)(PCy_3)$_2$].

	**193 K**
K	1.3 ± 0.1
**$\Delta\delta_{fit}$**	18.7

4. Stack Plots of ^{19}F NMR spectra

4.1. Titration plot of trans-[NiF(2-NC$_5$F$_3$H)(PEt$_3$)$_2$] with C$_6$F$_5$I

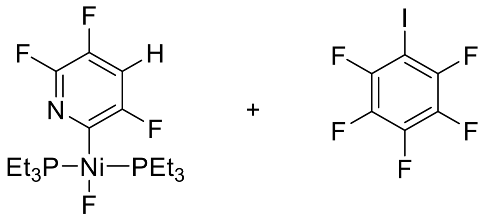

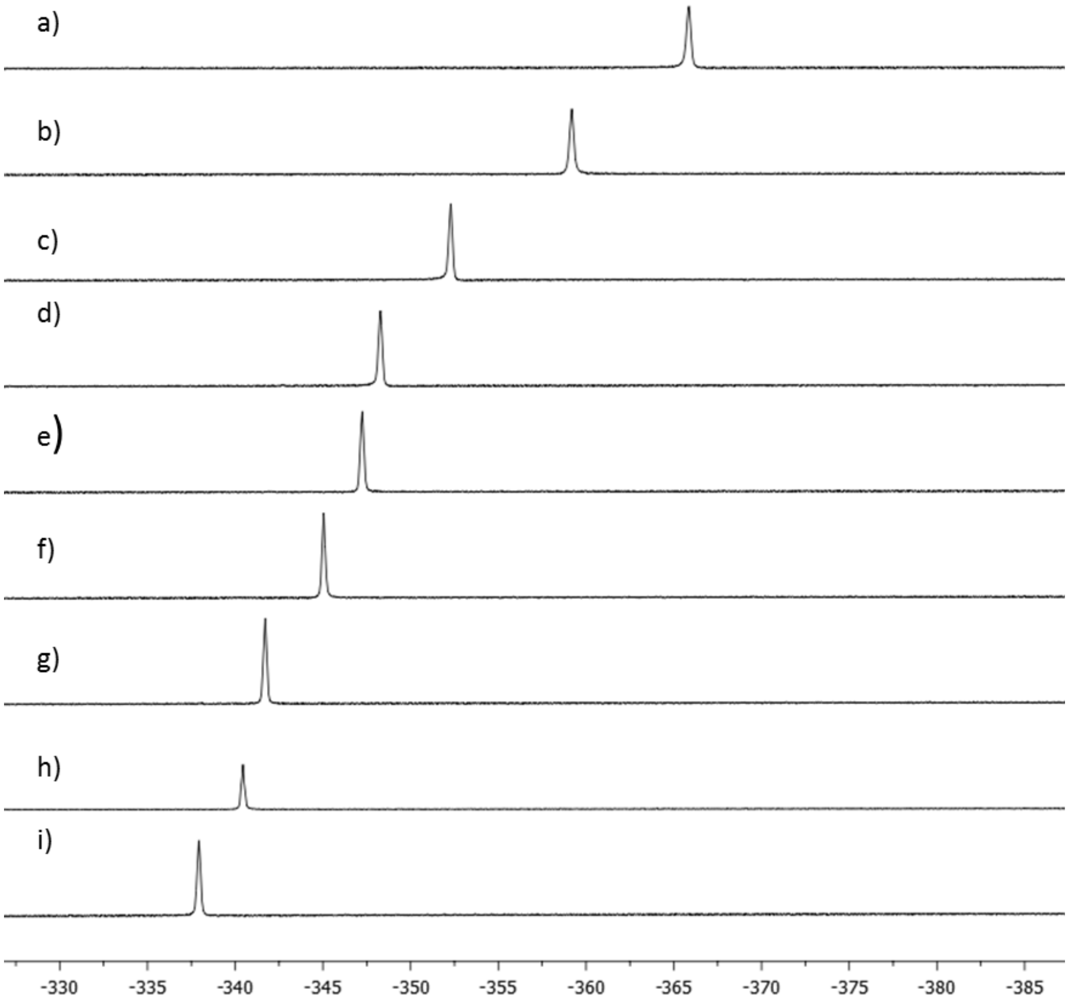

Figure S9. Stack plot of partial ^{19}F NMR spectra (toluene-h$_8$, 246 K), at different molar ratios of [C$_6$F$_5$I] / *trans*-[NiF(2-NC$_5$F$_3$H)(PEt$_3$)$_2$]. a) 0, b) 0.8, c) 2.1, d) 4.2, e) 4.6, f) 6.0, g) 9.7, h) 12.3, i) 18.6.

4.2. Titration plot of trans-[NiF{2-NC_5F_3(4-NMe_2)}(PEt_3)$_2$] with C_6F_5I

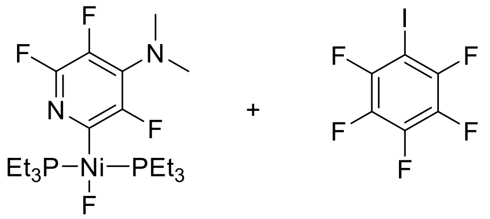

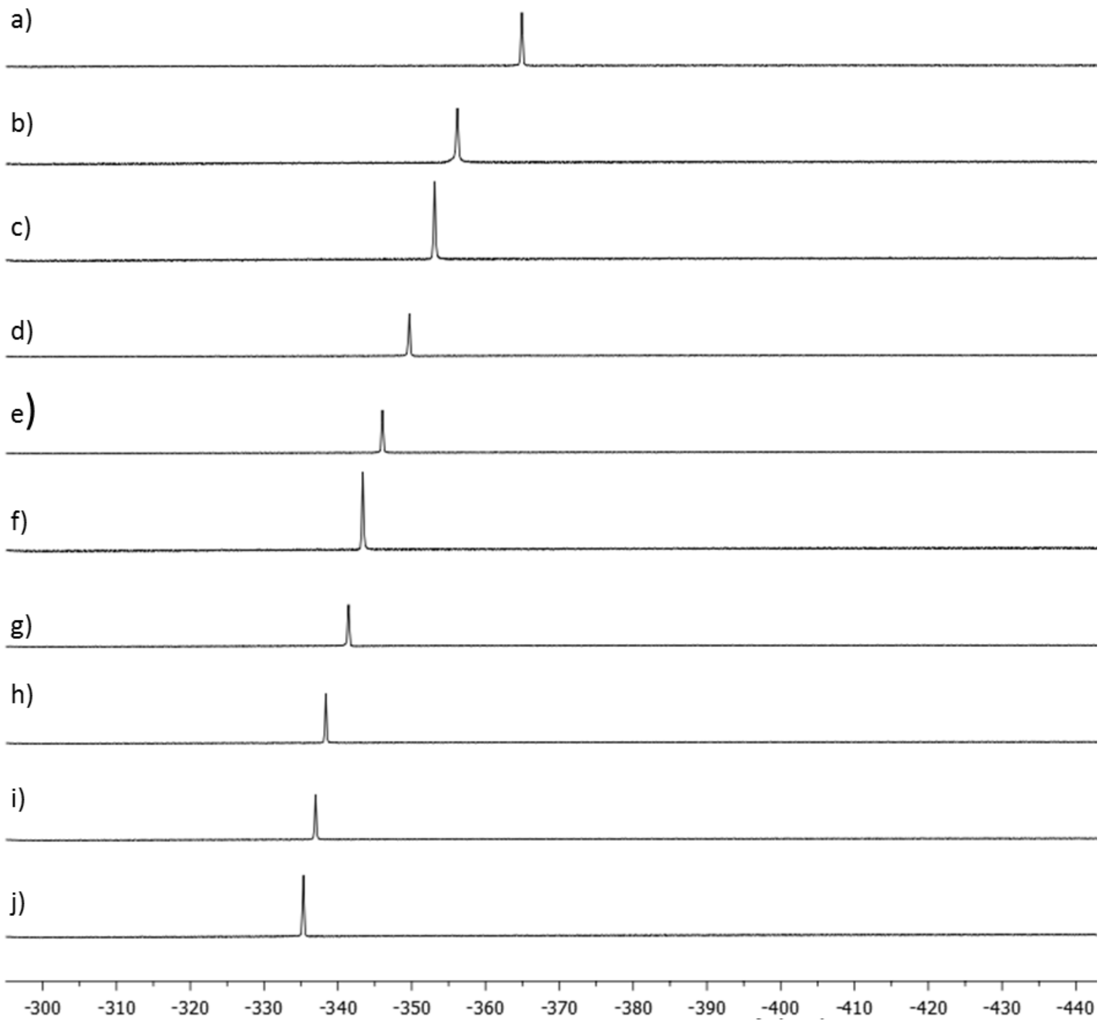

*Figure S10***.** Stack plot of partial ^{19}F NMR spectra (toluene-h_8, 246 K), at different molar ratios of [C_6F_5I] / *trans*-[NiF{2-NC_5F_3(4-NMe_2)}(PEt_3)$_2$]. a) 0, b) 0.92, c) 1.3, d) 1.8, e) 2.6, f) 3.8, g) 4.9, h) 8.1, i) 10.2, j) 14.5.

4.3. Titration plot of trans-[NiF(2-NC$_5$F$_3$H)(PEt$_3$)$_2$] with C$_4$F$_9$I

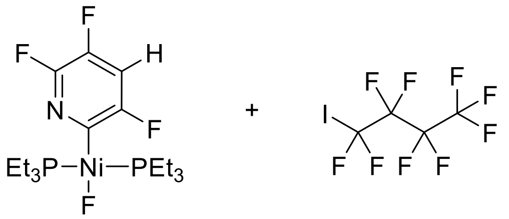

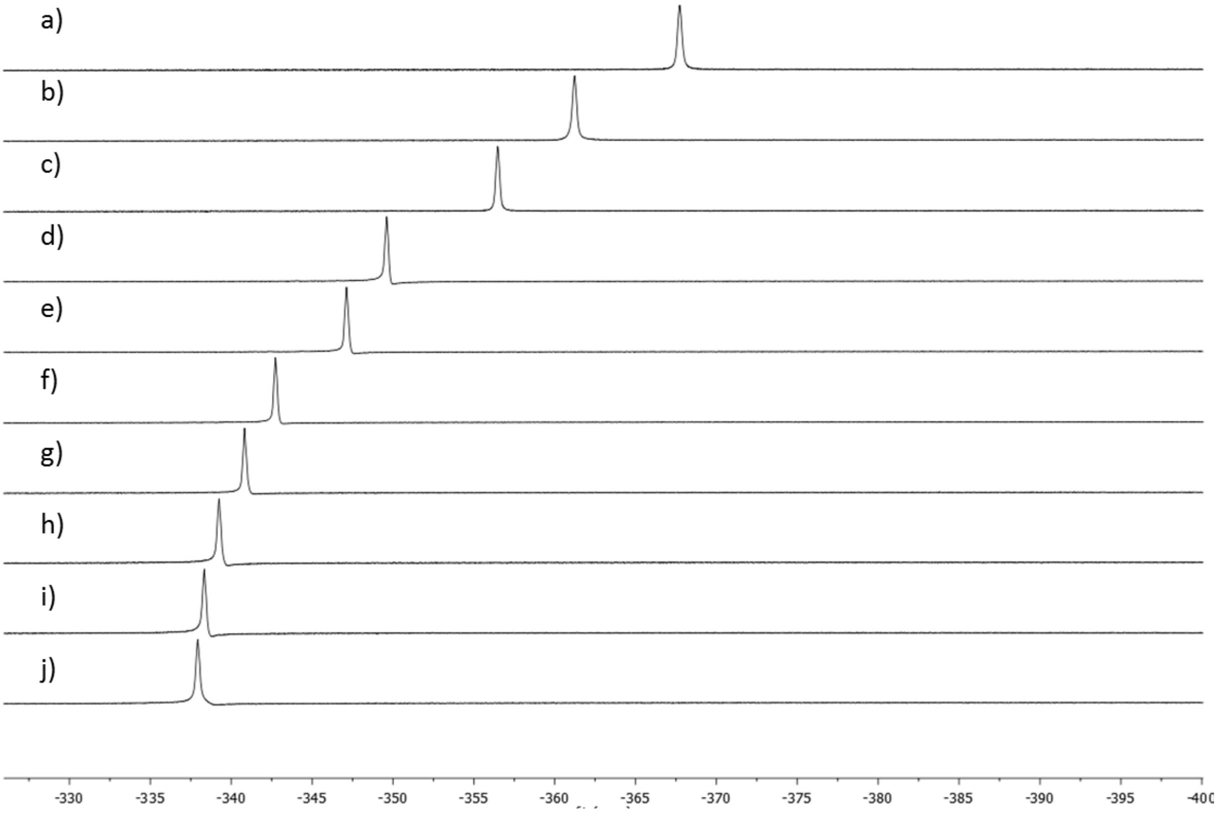

Figure S11. Stack plot of partial ^{19}F NMR spectra (toluene-h$_8$, 257 K), at different molar ratios of [C$_4$F$_9$I] / *trans*-[NiF(2-NC$_5$F$_3$H)(PEt$_3$)$_2$]. a) 0, b) 0.4, c) 0.8, d) 2.1, e) 2.6, f) 4.8, g) 6.7, h) 9.7, i) 12.8, j) 13.9.

4.4. Titration plot of trans-[NiF{2-NC_5F_3(4-NMe_2)}(PEt_3)$_2$] with C_4F_9I

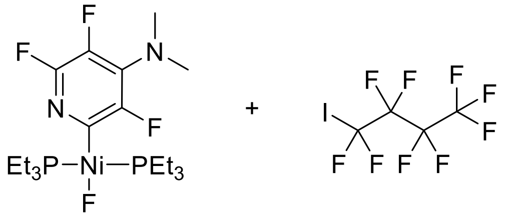

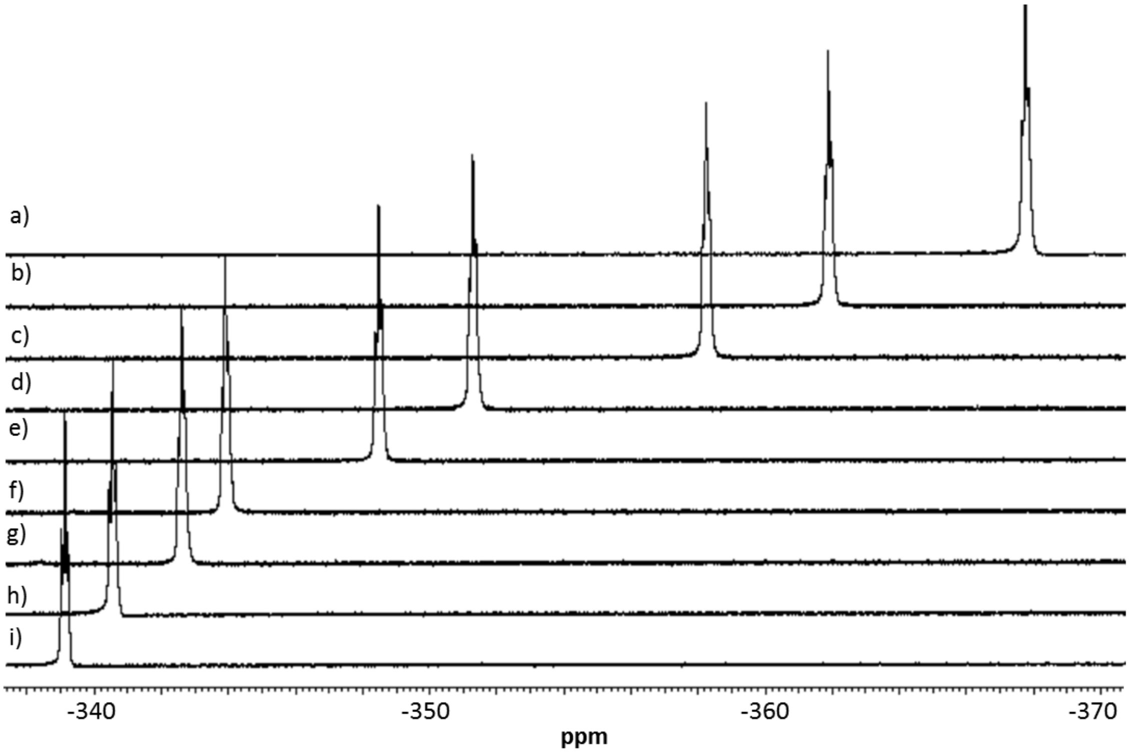

Figure S12. Stack plot of partial ^{19}F NMR spectra (toluene-h_8, 246 K), at different molar ratios of [C_4F_9I] / *trans*-[NiF{2-NC_5F_3(4-NMe_2)}(PEt_3)$_2$]. a) 0, b) 0.6, c) 1, d) 2.3, e) 3.3, f) 5.9, g) 7.1, h) 10.6, i) 15.3.

4.5. Titration plot of trans-[NiF(2-NC$_5$F$_3$H)(PEt$_3$)$_2$] with C$_6$F$_5$Br

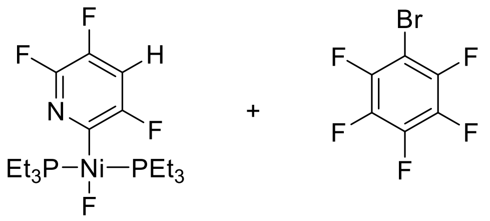

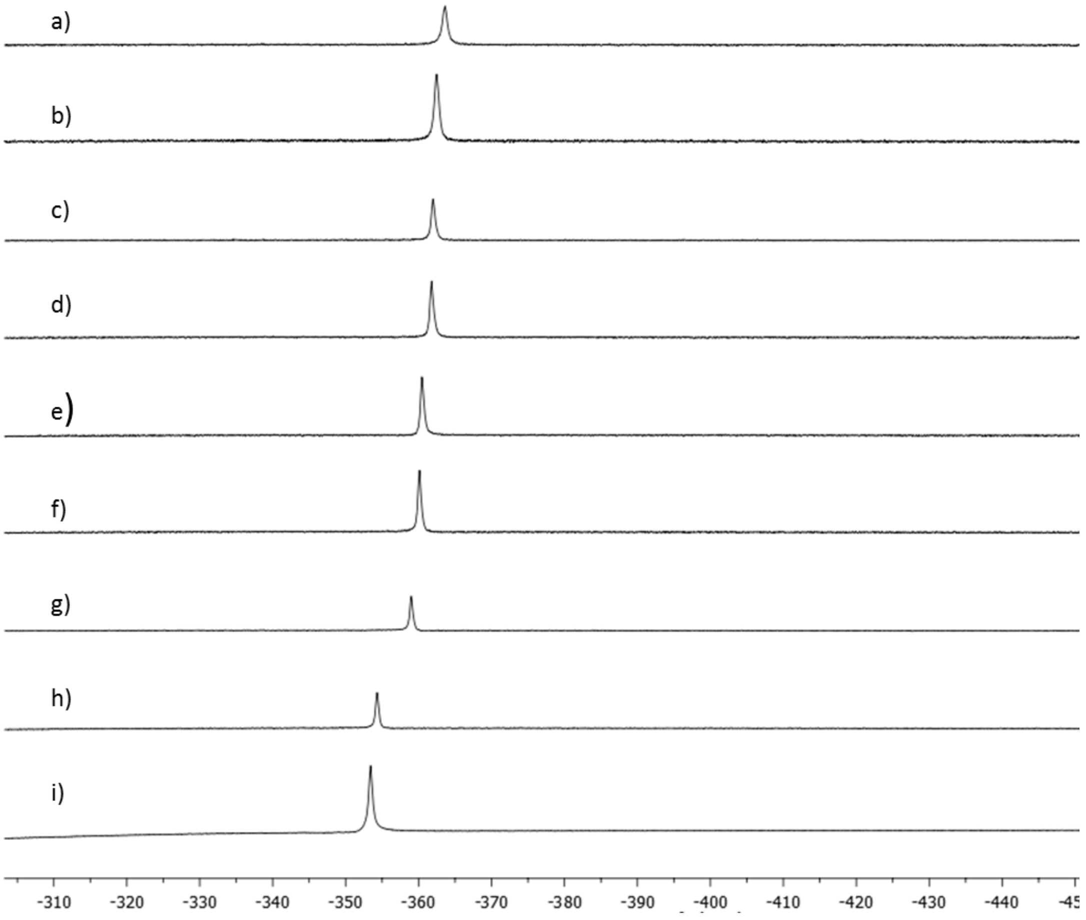

Figure S13. Stack plot of partial ^{19}F NMR spectra (toluene-h$_8$, 193 K), at different molar ratios of [C$_6$F$_5$Br] / *trans*-[NiF(2-NC$_5$F$_3$H)(PEt$_3$)$_2$]. a) 0, b) 4.5, c) 9.5, d) 12, e) 19, f) 22, g) 27, h) 92, i) 144.

4.6. Titration plot of trans-[NiF{2-NC$_5$F$_3$(4-NMe$_2$)}(PEt$_3$)$_2$] with C$_6$F$_5$Br

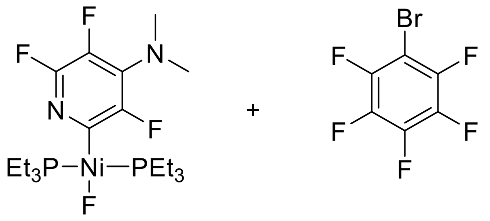

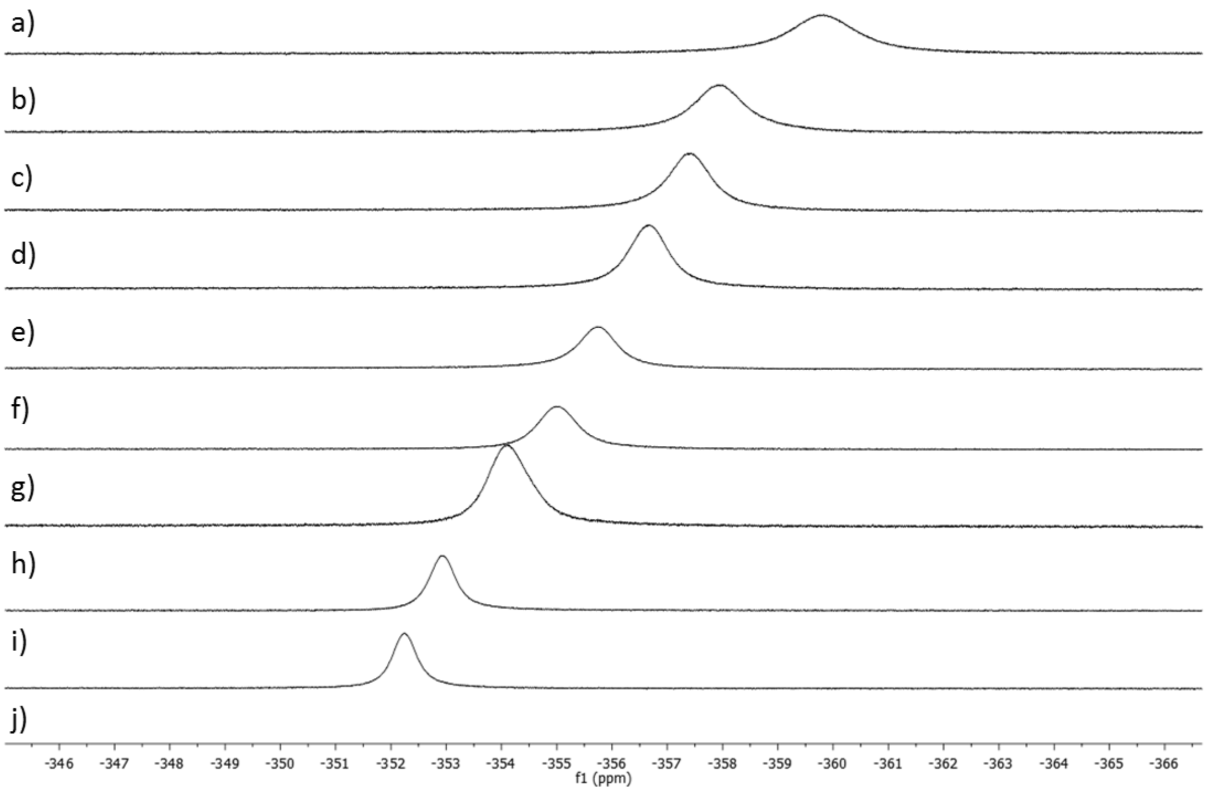

Figure S14. Stack plot of partial ^{19}F NMR spectra (toluene-h$_8$, 193 K), at different molar ratios of [C$_6$F$_5$Br] / *trans*-[NiF{2-NC$_5$F$_3$(4-NMe$_2$)}(PEt$_3$)$_2$]. a) 0, b) 12.9, c) 35, d) 44.1, e) 58.3, f) 73.7, g) 114.7, h) 132.9, i) 146.1.

4.7. Titration plot of trans-[NiF(2-NC$_5$F$_2$HCF$_3$)(PCy$_3$)$_2$] with C$_6$F$_5$Br

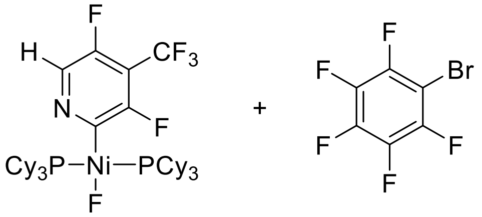

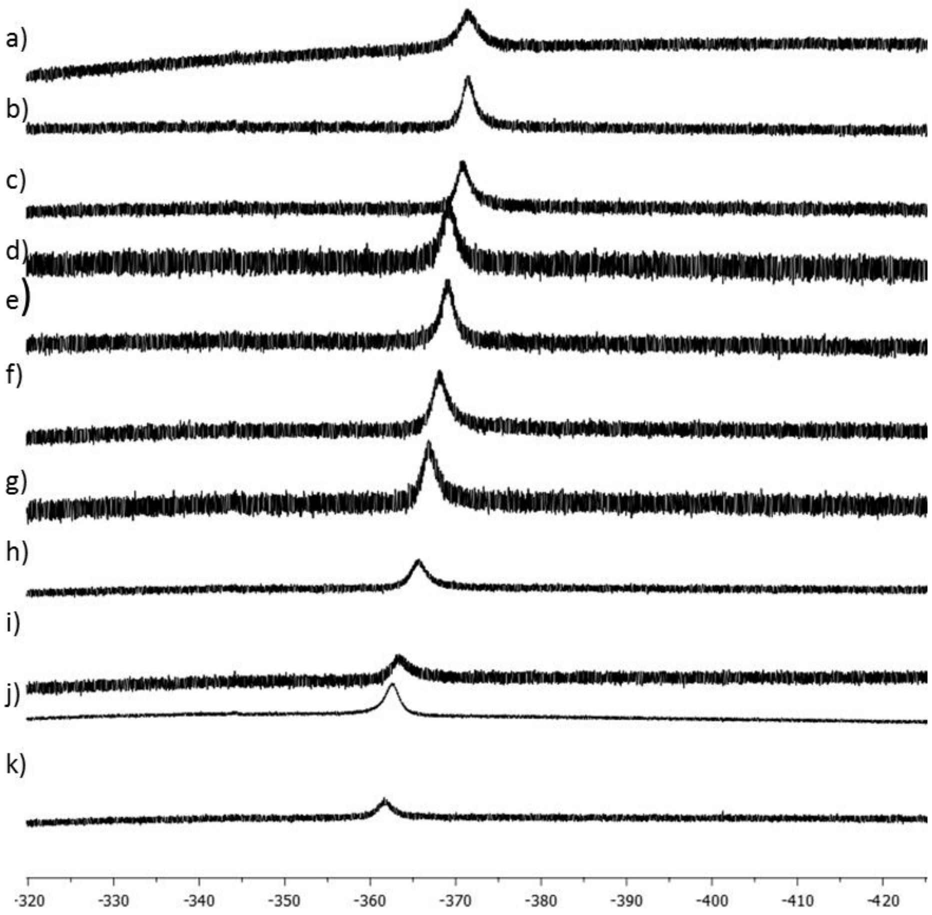

Figure S15. Stack plot of partial ^{19}F NMR spectra (toluene-h$_8$, 193 K), at different molar ratios of [C$_6$F$_5$Br] / *trans*-[NiF(2-NC$_5$F$_2$HCF$_3$)(PCy$_3$)$_2$]. a) 0, b) 8.9, c) 16.6, d) 30.7, e) 40.5, f) 48.9, g) 80, h) 101.8, i) 154, j) 202, k) 252.

5. Summary of Thermodynamic Parameters for Halogen Bonding Interactions

Table S10. Summary of thermodynamic parameters of halogen bonding interactions for metal fluorides **A2**-**A4**.

Halogen-bond donor	***Halogen-Bond Acceptor***	**Δ*H*° / kJ mol^{-1}**	**Δ*S*° / J mol^{-1} K^{-1}**
C_6F_5I (**D1**)	*trans*-[NiF(2-NC_5F_3H)(PEt_3)$_2$] (**A2**)	-18 ± 2	-46 ± 8
C_6F_5I (**D1**)	*trans*-[NiF{2-NC_5F_3(4-NMe_2)}(PEt_3)$_2$] (**A3**)	-17 ± 5	-39 ± 19
C_6F_5I (**D1**)	*trans*-[NiF(2-$NC_5F_2HCF_3$)(PCy_3)$_2$] (**A4**)	-19 ± 4[a]	-54 ± 1[a]
C_4F_9I (**D2**)	*trans*-[NiF(2-NC_5F_3H)(PEt_3)$_2$] (**A2**)	-23 ± 4	-53 ± 14
C_4F_9I (**D2**)	*trans*-[NiF{2-NC_5F_3(4-NMe_2)}(PEt_3)$_2$] (**A3**)	-22 ± 3	-50 ± 12
C_4F_9I (**D2**)	*trans*-[NiF(2-$NC_5F_2HCF_3$)(PCy_3)$_2$] (**A4**)	-19 ± 4	-51 ± 13

[a]Values from ref 3.

6. Temperature calibration of NMR spectrometer

Table S11. Nominal and calibrated (corrected) temperatures of NMR spectrometer.

**T (nom) / K**	**T(corr) / K**
212	192.7
223	205.1
233	222.7
243	233.6
253	246.2
263	259.3
273	271.0
283	284.8
293	294.2
298	297.6
300	299.8

7. References

1. C. Ammann, P. Meier, A. E. Merbach, *J. Magn. Reson.*, **1982**, *46*, 319-321.
2. T. A. Dransfield, R. Nazir, R. N. Perutz, A. C. Whitwood, *J. Fluor. Chem.*, **2010**, *131*, 1213-1217.
3. T. Beweries, L. Brammer, N. A. Jasim, J. E. McGrady, R. N. Perutz, A. C. Whitwood, *J. Am. Chem. Soc.*, **2011**, *133*, 14338-14348.
